# Gunshot vaginal trauma

**DOI:** 10.1016/j.radcr.2023.09.060

**Published:** 2023-10-17

**Authors:** Federica Dell'Aversana, Rosita Comune, Marco Scognamiglio, Francesca Grassi, Adele Durante, Roberta Avallone, Michele Tonerini, Pietro Affinito, Fabio Tamburro, Mariano Scaglione

**Affiliations:** a Division of Radiology, Università degli Studi della Campania Luigi Vanvitelli, Naples, Italy; b Department of Radiology, Ospedale del Mare-ASL NA1 Centro, Naples, Italy; c Department of Emergency Radiology, Cisanello Hospital, Pisa, Italy; d Department of Medicine, Surgery and Pharmacy, University of Sassari, Piazza Università, Sassari, Italy; e Department of Radiology, James Cook University Hospital, Middlesbrough, UK

**Keywords:** CT protocol trauma, Gunshot injury, Penetrating trauma, Vaginal trauma

## Abstract

Nonobstetric vaginal or vulva trauma is an extremely rare occurrence, with an incidence of < 0.2% of traumas**.** CT represents the gold standard in the diagnosis of gunshot lesions due to its ability to detect and stage injuries with very high sensitivity and specificity. A standardized protocol for penetrating trauma is still under debate for the use of intravenous contrast only or also rectal and oral contrast. Herein, we report a case of gunshot vaginal trauma in a 43-year-old patient presenting with vaginal bleeding. In our case, the protocol was “patient's tailored,” the intravaginal selective use of air was administered due to symptoms (vaginal bleeding) and CT findings, this 2-step protocol increased diagnostic confidence and allow a correct and challenging diagnosis.

## Introduction

Nonobstetric vaginal or vulva trauma is an extremely rare occurrence, with an incidence of < 0.2% of traumas [1]. Most penetrating injuries by gunshots or firearms occur in military settings [2,3]. In gunshot wounds, there is a high risk of visceral and abdominal vascular injuries associated with high morbidity and mortality. CT represents the gold standard in the diagnosis of gunshot lesions due to its ability to detect and stage injuries with very high sensitivity and specificity. CT can identify the exact bullet trajectory and the lesion related to the mechanics of gunshot injury (direct tissue damage with or without bleeding, cavitation, bone injuries) [4,5]. We report a case of gunshot vaginal trauma in a 43-year-old patient presenting with vaginal bleeding.

### Case presentation

A 43-year-old woman was referred to our institution in emergency room settings for a gunshot injury with an entry hole in the right gluteal region, the exit wound was not appreciable at physical examination. The patient was hemodynamically stable *(BP 130/70 mm Hg, HR= 95 bpm, saturation 99%, GCS = 14)* with significant vaginal hemorrhage.

Multidetector computer tomography of the abdomen and pelvis was performed with and without contrast. Nonenhanced, arterial, portal venous, and delayed phases were acquired.

**Fig. 1:**
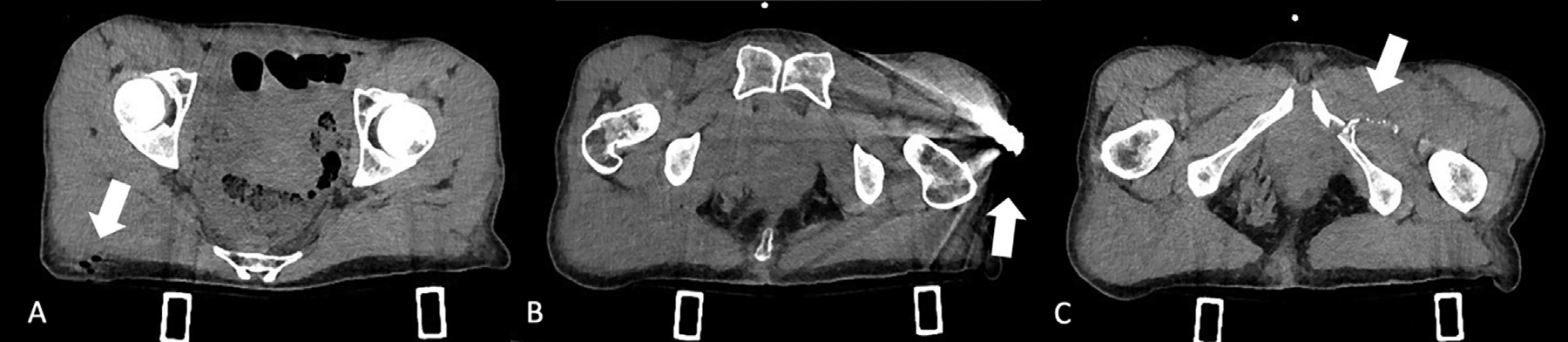
Axial CT scan during the pre-contrast phase showing (A) gunshot entry hole in the right gluteal region, (B) the retained bullet localized in the left coxofemoral subcutaneous region, (C) fragmented fracture of the left ischiopubic branch with evidence of multiple tiny bone pieces retained within the ipsilateral adductor muscles.

At CT examination, no intra-abdominal free air or hemoperitoneum was appreciable. The gunshot entry hole was detected in the right gluteal region and the retained bullet was localized in the left coxofemoral subcutaneous region with associated fragmented fracture of the left ischiopubic branch with evidence of multiple tiny bone pieces retained within the ipsilateral adductor muscles (Figs. 1A–C).

No signs of active bleeding or major vascular injury were appreciable. No penetrating lesions of the rectus, bladder, or uterine were detected.

**Fig. 2:**
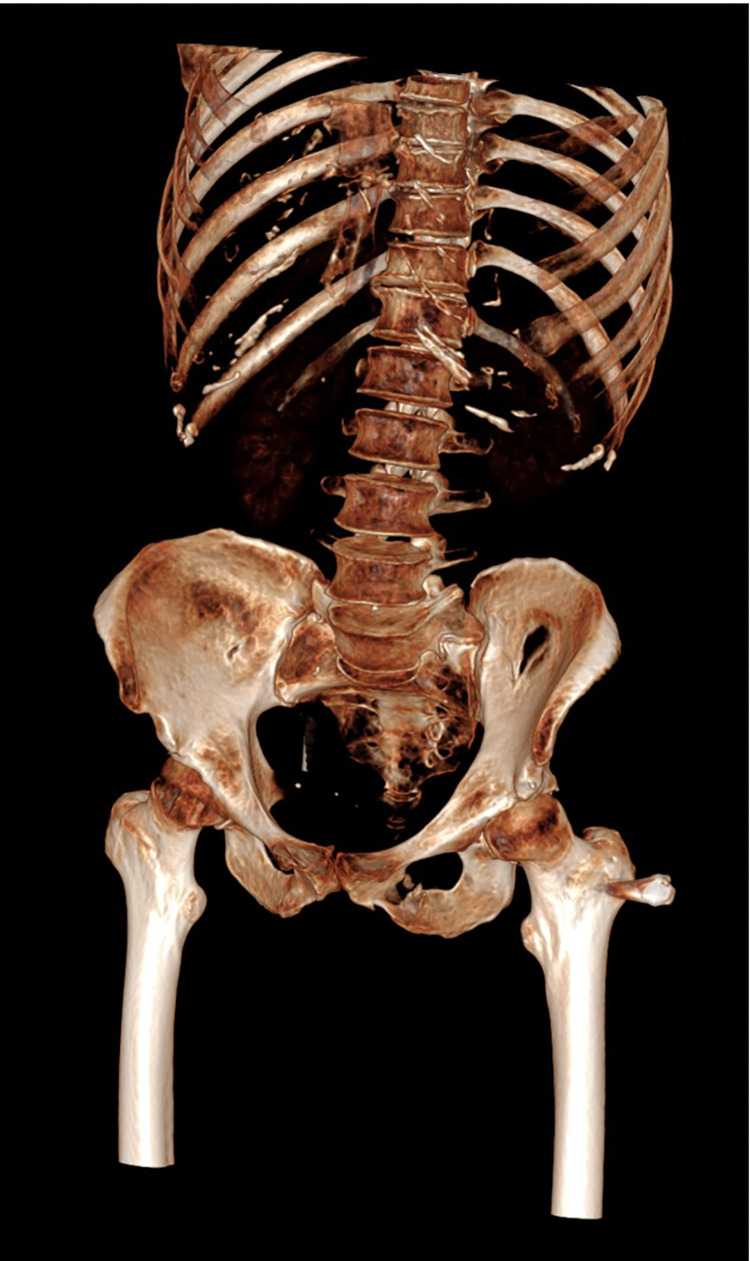
Coronal three-dimensional volume-rendered image demonstrates the path of the bullet trajectory the fragmented fracture of the left ischiopubic branch and the retained bullet.

**Fig. 3:**
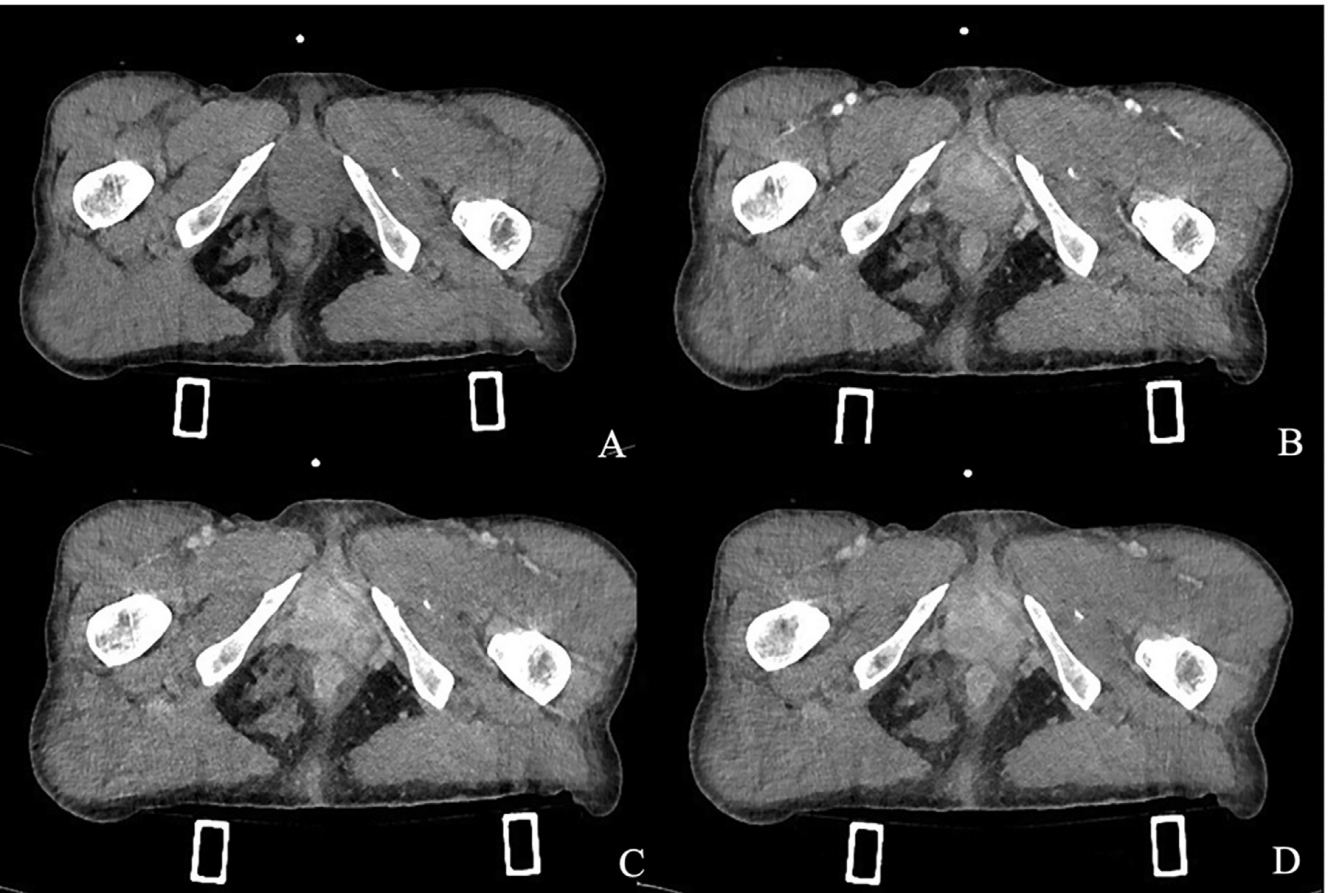
(A–C) CT axial scan showing phase without contrast (A), during the arterial phase (B), the nephrographic phase (C) and delayed phase (D) demonstrating suprafluid-blood distention of the vagina and thickening and imbibition of the adjacent soft tissues.

**Fig. 4:**
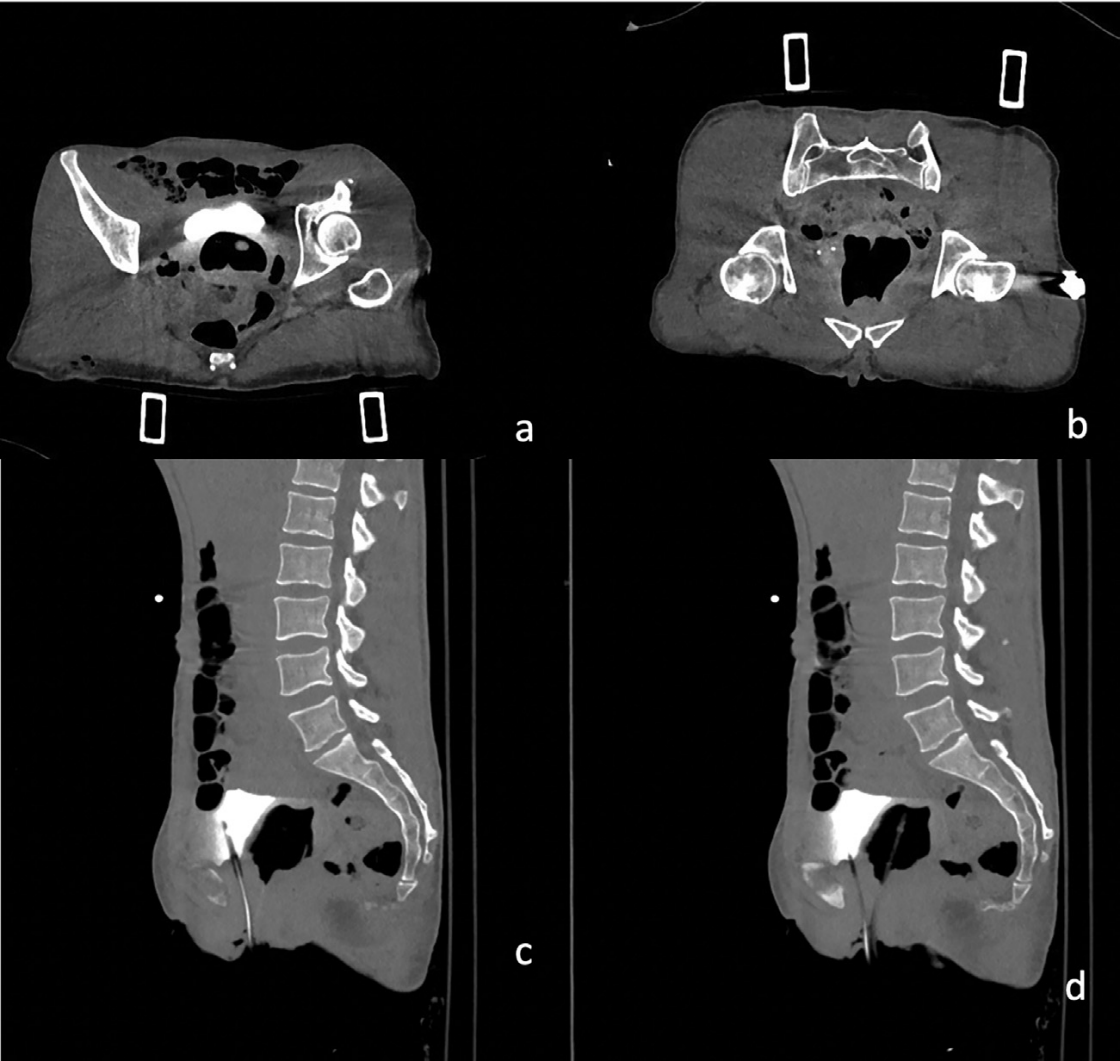
(A–D) CT axial and sagittal scan after the administration of Intravaginal positive contrast medium through a vaginal catheter demonstrates the extravasation of air and contrast confirming the diagnosis of a vaginal wound.

Following the bullet's path through the pelvis (Fig. 2), a suspected diagnosis of a wound tract through the vagina was formulated. The vagina appeared distended by blood, particularly at the vaginal fornixes; perivaginal soft tissues were thickened and hyperemic (Figs. 3A–D). Intravaginal negative contrast medium (air) was administered through a vaginal catheter and the previously described collection expanded, confirming the diagnosis of a vaginal wound tract (Figs. 4A–D). Gynecological examination and transvaginal ultrasound confirmed uterus integrity. The patient underwent surgical repair of the vagina. At the gynecological examination, 2 continuous bleeding solutions (about 1 cm in diameter) of the vaginal walls were detected and subsequently sutured under adequate analgesia. Also antibiotic prophylaxis and tetanus prophylaxis were carried out. Lesions were in the middle third of the vagina: the entry wound was located on the posterior wall, and the exit wound was cranial and located on the left posterolateral wall. Both wounds were sutured.

Subsequently, the bullet retained in the left subcutaneous region was extracted.

## Discussion

Penetrating trauma is a significant cause of morbidity and mortality worldwide and is secondary to a violation of the abdominal cavity by any object capable of penetrating the body: it comprises a wide range of mechanisms that can be divided into nonballistic (stab and puncture wounds) and ballistic trauma (gunshot wounds) [6]. Firearm injuries are responsible for about 7% of premature deaths before the age of 65 [5]. Penetrating trauma can result in life-threatening injury due to massive hemorrhage, and organ disruption.

Hence the importance of the CT study given its ability to detect injuries with very high sensitivity and specificity and to characterize the bullet's trajectory. CT permit to identify the trajectory of the bullet through the body in order to understand where the greatest amount of kinetic energy is transferred. In fact, the deposition of the projectile energy has various effects in different tissues: if the elastic limit of the tissue is exceeded, the soft tissues tear, the bones fracture or can make the projectile ricochet creating another wound tracks for the fragmentation of the bullet. The alterations affecting the tissue encountered by the projectile depend on its mass, on the speed of the impact, on the type of tissue encountered, on the composition and construction of the projectile and on the path of the projectile [5].

Gunshot wounds in the pelvis can inflict injuries on multiple organs and vessels simultaneously, making it therefore essential to study the bullet's trajectory in order to estimate the extent of the damage and establish the most appropriate therapeutic approach.

Penetrating gluteal trauma is a common cause of admission to trauma centers, although the real incidence is unknown. Lunevicius et al., in an analytical review of 664 cases of penetrating buttock trauma, demonstrated that 95.4% of patients were young males and that bleeding and penetrating lesions of small bowel, colon, or rectum were the commonest lesions [7,8]. A recent systematic review attempted to fill the gap that exists in the literature on penetrating injuries to the perineum and associated pelvic organs in the civilian setting, demonstrating that the anus-rectum, bladder, and scrotum are frequently injured with associated vascular injuries reported in 7.8% of patients [9]. None of these studies investigated vaginal injuries, which are extremely rare in the context of penetrating gunshot trauma that typically affects young men [8].

CT diagnosis of penetrating trauma can be challenging, it's fundamental for the radiologist to be familiar with projectile kinetics in order to identify both subtle and overt injuries. A useful tool in CT interpretation of penetrating trauma is CT *trajectography*, this tool is based on the connection of the entry and exit wounds by a placing cross-cursor, and using MPR reconstruction it's possible to cut planes obliquely in orthogonal planes obtaining a double-oblique orientation along bullet's trajectory [5]. In order to predict which organs are most likely to be injured, it is therefore useful to divide the human body into regions using superficial landmarks to identify and extrapolate the possible injury trajectory [10].

In our case, at physical examination, the entry wound was in the right gluteal region with loss of cutaneous and subcutaneous substances. At CT, the entry wound was characterized by edema and hemorrhagic infarction of subcutaneous planes and of the gluteus maximus muscle and by the presence of air bubbles. There was also evidence of hemorrhagic infarction in the right ischio-rectal fossa. The retained bullet was localized in the left coxofemoral subcutaneous with an associated fragmented fracture of the left ischiopubic.

Tracing the bullet's path through the pelvis, the suspicion of vaginal laceration was raised due to the evidence of a bloody distended vagina more evident and the thickening of the perivaginal soft tissues. Intravaginal negative contrast (air) was administered and increased diagnostic confidence for the diagnosis of vaginal laceration.

Our experience supports what was previously published [5], because a standardized protocol for penetrating trauma for the use of intravenous contrast alone or also of rectal and oral contrast is still under discussion. In our case, a "tailor-made" protocol was carried out for the patient, selective intravaginal use of air was administered for the CT results, this 2-step protocol increased diagnostic confidence and allowed for a correct diagnosis and challenging.

## Conclusion

Nonobstetric vulvovaginal lacerations are underreported and are extremely rare, an isolated gunshot laceration of the vagina represents an exceptional event. Conservative treatment is usually recommended in women with minor injuries but female patients suffering from gunshot wounds to the pelvic area should be always investigated for vaginal, urethral, anal, and bony pelvis injuries [5,11]. It's fundamental to be familiar with projectile kinetics to extrapolate bullet's trajectory, CT trajectography can be extremely useful to identify lesion. Gynecological examination allows to identify signs of involvement of the internal genitalia and is a fast and inexpensive method. However, in the hemodynamically stable patient undergoing radiological investigations, a patient's tailored protocol with the administration of intravaginal contrast medium can provide additional information, increase diagnostic confidence and can be used as problem-solving tool after performing triple-phase CT with intravenous contrast material.

## Patient consent

Written informed consent was obtained from the patient for publication of this case report and accompanying images. A copy of the written consent is available for review by the Editor-in-Chief of this journal on request.
